# Pericardial fat, thoracic peri-aortic adipose tissue, and systemic inflammatory marker in nonalcoholic fatty liver and abdominal obesity phenotype

**DOI:** 10.1038/s41598-022-06030-z

**Published:** 2022-02-04

**Authors:** Chun-Ho Yun, Jing-Rong Jhuang, Meng-Ting Tsou

**Affiliations:** 1 MacKay Junior College of Medicine, Nursing, and Management, New Taipei, Taiwan; 2 Department of Radiology, MacKay Memorial Hospital, Taipei, Taiwan; 3 Institute of Epidemiology and Preventive Medicine, College of Public Health, National Taiwan University, Taipei, Taiwan; 4 Department of Family Medicine, MacKay Memorial Hospital, No. 92, Sec. 2, Zhongshan N. Rd, Taipei City, 10449 Taiwan Republic of China; 5 Department of Occupation Medicine, Mackay Medical College, Taipei, Taiwan

**Keywords:** Cardiovascular biology, Cardiology

## Abstract

Researchers have conducted many studies about the relationships between peri-cardiovascular fat, nonalcoholic fatty liver disease (NAFLD), waist circumference, and cardiovascular disease (CVD). Nevertheless, the relationship between NAFLD and pericardial fat (PCF)/thoracic peri-aortic adipose tissue (TAT) phenotypes was still unknown. This study aimed to explore whether PCF/TAT was associated with NAFLD/abdominal obesity (AO) phenotypes in different high-sensitivity C-reactive protein (hs-CRP) levels. We consecutively studied 1655 individuals (mean age, 49.44 ± 9.76 years) who underwent a health-screening program. We showed a significant association between PCF/TAT and NAFLD/AO phenotypes in the cross-sectional study. We observed that the highest risk occurred in both abnormalities’ groups, and the second highest risk occurred in the AO-only group. Subjects with AO had a significantly increased risk of PCF or TAT compared to those with NAFLD. Notably, the magnitude of the associations between PCF/TAT and NAFLD/AO varied by the level of systemic inflammatory marker (hs-CRP level). We suggested that people with AO and NAFLD must be more careful about changes in PCF and TAT. Regular measurement of waist circumference (or AO) can be a more accessible way to monitor peri-cardiovascular fat (PCF and TAT), which may serve as a novel and rapid way to screen CVD in the future.

## Introduction

Over-production of various systemic inflammatory mediators by excessive adipose tissue that leads to a variety of metabolic disorders^1,2^. Previous studies mentioned that two volume-based measures of region-specific visceral adipose tissue, epicardial fat (EAT) including adipose tissue inside pericardium and along myocardium as well as thoracic peri-aortic adipose tissue (TAT) including adipose tissue confined in the area along the margin of thoracic vertebral body, and surrounding thoracic descending aorta—have been shown to correlate with metabolic risk factors and atherosclerosis^3,4^, which are independently associated systemic inflammatory markers such as hs-CRP^5,6^.

As we know, abdominal and visceral obesity have been considered as major risk factors for coronary artery disease (CAD)^7^. Waist circumference (WC) allows visceral adiposity to be differentiated from total obesity, correlates well with abdominal imaging findings, and is more predictive of coronary artery calcification (CAC) than other anthropometric indicators^8^. It has been widely accepted as an indicator of poor metabolism and high cardiovascular risk^9^. Many studies have focused on abdominal fat tissue closely related to epicardial adiposity tissue (EAT)^4,9^. It has also been confirmed that the thickness of EAT is closely related to Waist circumference (WC)^9^. On the other hand, in the past decade, the terminology of adipose tissue close to heart is heterogeneous and inconsistent. Recently, more and more researchers used EAT to represent adipose inside pericardial sac. In addition, some researchers measured intrathoracic fat combined EAT and all adipose tissue in thoracic cage but outside pericardium and demonstrated a significant association with incidence of cardiovascular disease and LV functional deterioration^10–13^. Anatomically, EAT is adjacent to myocardium directly but adipose tissue located outside but proximity to pericardium is still very close to heart. Therefore, we used the definition of PCF as EAT plus paracardial adipose tissue which is close to but outside pericardium and investigated the relationship between PCF/TAT, these two regional-specific adipose tissues and WC/other visceral adipose tissue.

Nonalcoholic fatty liver disease (NAFLD) is defined as the accumulation of more than 5% fat in hepatocytes in individuals whose alcohol intake is lower than 20 g/day^14^. It has become a common health problem with a prevalence of approximately 20–30% in the Western population^15^; among extensive population-based surveys in Asia, the prevalence rates of NAFLD were between 2.04 and 52%^16,17^. Taiwan has a prevalence of 11.5–52%^18^. NAFLD is closely related to liver metabolic syndrome, obesity, inflammation markers and insulin resistance (IR)^19–21^. In addition, patients with NAFLD patients experience early-onset cardiovascular changes^22^; therefore considered an independent risk factor for cardiovascular disease (CVD)^23^. In a recent Chinese population study on NAFLD and CVD, it has demonstrated the association of EAT with NAFLD and CVD^24^. Whether NAFLD could affect other visceral adipose tissue around cardiovascular system, such as PCF and TAT has not been studied extensively.

This study aimed to explore the association between PCF/TAT and abdominal obesity (AO)/NAFLD. The confirmation of the association contributes to a more accessible way (WC and NAFLD) to monitor changes in peri-cardiovascular fat (PCF and TAT), which may serve as a novel and rapid way to screen CVD in the future. In addition, to find out whether there is a correlation with the inflammation index (hs-CRP).

## Results

A total of 1655 participants were included for analysis, including 1164 men (70.3%, mean age 48.67 ± 9.61 years) and 491 women (29.7%, 51.26 ± 9.88 years). Among all the participants, 995 (60.1%) had NAFLD and 605 (36.6%) had AO. Both NAFLD and AO prevalence increased with PCF and TAT tertile (Supplementary Fig. 1). A similar trend was observed for visceral adiposity (p < 0.001). The participants were categorized according to NAFLD/AO phenotype as follows: (1) subjects without either abnormality (n = 545, 32.9%); (2) subjects with AO only (n = 115, 7.0%); (3) subjects with NAFLD only (n = 505, 30.5%), and (4) subjects with both abnormalities (n = 490, 29.6%).

Table 1 presented the comparison of factors between the NAFLD/AO phenotypes. The AO-only group was the oldest, and both abnormalities group had the worst metabolic factor results. The NAFLD-only group had a worse lipid profile than that of the AO-only group. The mean WC, WC/hip ratio, and WC/height ratio for all participants were 84.45 ± 10.07 cm, 0.89 ± 0.07, and 0.51 ± 0.06, respectively. Compared with the NAFLD-only group, the AO-only group had a higher mean BMI, WC/hip ratio, and WC/height ratio, with a higher proportion of obese individuals. The both abnormalities group had the highest proportion of subjects with abnormal liver function test results, diabetes mellitus (DM), dyslipidemia, PCF, and TAT. The proportions of subjects with DM, HTN, dyslipidemia, PCF, and TAT in the AO-only group were higher than those in the NAFLD-only group. The mean PCF and TAT were higher in the group with both abnormalities. The AO-only group had a higher proportion with moderate/high hs-CRP level than that of the NAFLD-only group.

**Table 1:** Baseline characteristics between the groups according to NAFLD and AO status.

Variables	Total population	NAFLD (−) AO (−)	NAFLD (−) AO (+)	NAFLD (+) AO (−)	NAFLD (+) AO (+)	*p* value
	(n = 1655)	(n = 545)	(n = 115)	(n = 505)	(n = 490)	
**Baseline characters**						
Age (year)	49.44 ± 9.76	48.20 ± 9.71	54.22 ± 10.97^a^	48.03 ± 8.72^b^	51.15 ± 9.92^a,b,c^	< 0.001
Gender (male), n (%)	1164 (70.30%)	347 (63.70%)	62 (53.90%)	404 (80.00%)^a,b^	351 (71.60%)^a,b,c^	< 0.001
BMI (kg/m^2^)	24.66 ± 3.57	22.04 ± 2.22	26.16 ± 3.05^a^	23.83 ± 2.07^a,b^	28.08 ± 3.23^a,b,c^	< 0.001
WC (cm)	84.45 ± 10.07	77.01 ± 7.08	90.19 ± 7.19^a^	81.52 ± 5.63^a,b^	94.40 ± 8.07^a,b,c^	< 0.001
WC/hip ratio	0.89 ± 0.07	0.85 ± 0.06	0.91 ± 0.05^a^	0.88 ± 0.05^a,b^	0.94 ± 0.06^a,b,c^	< 0.001
WC/height ratio	0.51 ± 0.06	0.47 ± 0.04	0.55 ± 0.04^a^	0.49 ± 0.03^a,b^	0.57 ± 0.05^a,b,c^	< 0.001
SBP (mmHg)	122.27 ± 17.00	118.06 ± 16.47	125.68 ± 17.22^a^	120.12 ± 15.33^b^	128.36 ± 17.33^a,c^	< 0.001
DBP (mmHg)	76.77 ± 10.54	73.41 ± 10.02	78.60 ± 10.32^a^	76.02 ± 10.17^a^	80.85 ± 10.11^a,c^	< 0.001
**Lab data**						
FPG (mg/dL)	102.05 ± 23.98	96.49 ± 17.74	101.14 ± 20.87	99.24 ± 19.59	111.35 ± 31.12^a,b,c^	< 0.001
TC (mg/dL)	203.00 ± 37.82	197.48 ± 35.84	198.32 ± 34.22	204.21 ± 40.25^a^	208.99 ± 37.28^a,b^	< 0.001
TG (mg/dL)	115 [82, 170]	90 [66.0, 120.5]	106 [83.0, 149.0]^a^	119 [88.5, 170.0]^a^	154.5 [108.0, 221.0]^a,b,c^	
HDL-C (mg/dL)	52.55 ± 14.18	58.97 ± 14.90	52.47 ± 13.81^a^	51.16 ± 13.04^a^	46.86 ± 11.51^a,b,c^	< 0.001
LDL-C (mg/dL)	131.70 ± 32.90	125.58 ± 32.27	128.13 ± 31.54	134.53 ± 32.52^a^	136.4 ± 33.24^a^	< 0.001
AST (IU/L)	25.26 ± 14.33	22.49 ± 11.66	24.35 ± 11.08	24.55 ± 10.03	29.28 ± 19.62^a,b,c^	< 0.001
ALT (IU/L)	31.77 ± 28.75	23.87 ± 26.13	26.56 ± 15.64	31.76 ± 21.49^a^	41.72 ± 36.41^a,b,c^	< 0.001
**Medical history**						
Smoking, n (%)	230 (13.90%)	56 (10.30%)	12 (10.40%)	81 (16.00%)^a^	81 (16.50%)^a^	0.01
Alcohol drinking, n (%)	103 (6.20%)	24 (4.40%)	5 (4.30%)	31 (6.10%)	43 (8.80%)^a^	0.03
Exercise, n (%)	277 (16.70%)	92 (16.90%)	22 (19.10%)	91 (18.00%)	72 (14.70%)	0.47
HTN, n (%)	309 (18.70%)	63 (11.60%)	36 (31.30%)^a^	70 (13.90%)^b^	140 (28.60%)^a,c^	< 0.001
DM, n (%)	110 (6.60%)	26 (4.80%)	9 (7.80%)	22 (4.40%)	53 (10.80%)^a,c^	< 0.001
Hyperlipidemia, n (%)	100 (6.00%)	19 (3.50%)	8 (7.00%)	26 (5.10%)	47 (9.60%)^a,c^	< 0.001
**Visceral fat burden**						
PCF (ml)	75.82 ± 31.90	59.77 ± 23.44	83.94 ± 33.97^a^	73.77 ± 26.45^a,b^	93.88 ± 34.73^a,b,c^	< 0.001
1rd tertile, n (%)	551 (33.29%)	302 (55.41%)	23 (20.00%)^a^	169 (33.47%)^a,b^	57 (11.63%)^a,c^	
2rd tertile, n (%)	552 (33.35%)	176 (32.29%)	39 (33.91%)	179 (35.45%)	158 (32.24%)	< 0.001
3rd tertile, n (%)	552 (33.35%)	67 (12.29%)	53 (46.09%)^a^	157 (31.09%)^a,b^	275 (56.12%)^a,c^	
TAT (ml)	6.90 ± 3.89	4.87 ± 2.76	7.88 ± 4.26^a^	6.62 ± 2.82^a,b^	9.23 ± 4.48^a,b,c^	< 0.001
1rd tertile, n (%)	551 (33.29%)	319 (58.53%)	30 (26.09%)^a^	133 (26.34%)^a^	69 (14.08%)^a,b,c^	
2rd tertile, n (%)	551 (33.29%)	150 (27.52%)	41 (35.65%)	221 (43.76%)^a^	139 (28.37%)^c^	< 0.001
3rd tertile, n (%)	553 (33.41%)	76 (13.94%)	44 (38.26%)^a^	151 (29.90%)^a^	282 (57.55%)^a,b,c^	
**Inflammation marker**						
hs-CRP (md/dL)	0.19 ± 0.34	0.13 ± 0.34	0.26 ± 0.40^a^	0.17 ± 0.28^a,b^	0.27 ± 0.36^a,b,c^	< 0.001
Moderate/high, n (%)	830 (50.16%)	163 (29.93%)	70 (61.02%)^a^	237 (46.98%)^a^	355 (72.47%)^a^	< 0.001
Low, n (%)	825 (49.84%)	382 (70.07%)	45 (38.98%)^a^	268 (53.02%)^a^	135 (27.53%)^a^	

Abbreviations: *NAFLD* non-alcoholic fatty liver disease, *AO* abdominal obesity, *BMI* body mass index, *WC* waist circumference, *SBP* systolic blood pressure, *DBP* diastolic blood pressure, *FPG* fasting plasma glucose, *TC* total cholesterol, *TG* triglyceride, *HDL-C* high density lipoprotein cholesterol, *LDL-C* low density lipoprotein cholesterol, *AST* aspartate aminotransferase, *ALT* alanine aminotransferase, *HTN* hypertension, *DM* diabetes mellitus, *PCF* pericardial fat, *TAT* thoracic peri-aortic adipose tissue, *hs-CRP* high-sensitivity C-reactive protein.

^a^*p* < 0.05 versus NAFLD (−) Abdominal obesity (−).

^b^*p* < 0.05 versus NAFLD (−) Abdominal obesity (+).

^c^*p* < 0.05 versus NAFLD (+) Abdominal obesity (−) in the Bonferroni post hoc comparisons.

Table 2 presented the results of the multiple linear regression stratified by hs-CRP subgroup. In the univariate analyses, all *β*s were greater than zero and statistically significant, which means that there was a positive association between PCF/TAT and NAFLD/AO phenotype. After adjusting for age, sex, DM, HTN, hyperlipidemia history, cigarette smoking, alcohol drinking, and exercise, the strength of the association became slightly weaker but remained statistically significant. For the moderate/high hs-CRP group, the correlation between AO and PCF/TAT was greater than with NAFLD (PCF: standardized *β* = 0.24 for AO and 0.11 for NAFLD; TAT: standardized *β* = 0.11 for AO and NAFLD). Notably, supra-additive effects were observed (PCF: standardized *β* = 0.45 for the abnormalities group [> 0.35 = 0.24 + 0.11]; TAT: standardized *β* = 0.40 for the abnormalities group [> 0.11 + 0.11]). For the low hs-CRP group, the correlation between NAFLD and PCF/TAT was greater than fore AO (PCF: standardized *β* = 0.23 for NAFLD and 0.08 for AO; TAT: standardized *β* = 0.22 for NAFLD and 0.15 for AO). A supra-additive effect was observed only for PCF (standardized *β* = 0.40 for the abnormalities group [> 0.23 + 0.08]).

**Table 2:** Results of the multiple linear regression stratified by hs-CRP subgroup.

Variables	NAFLD (−) abdominal obesity (−)			NAFLD (−) abdominal obesity (+)			NAFLD (+) abdominal obesity (−)			NAFLD (+) abdominal obesity (+)		
	β	(95% CI)	*p* value	β	(95% CI)	Std. β	β	(95% CI)	Std. β	β	(95% CI)	Std. β
**Moderate/high hs-CRP group**												
PCF												
Model 1	(Reference)			31.60	(19.78–43.42)	0.25	9.79	(1.60–17.97)	0.13	34.32	(26.78–41.87)	0.51
Model 2				30.66	(19.37–41.94)	0.24	8.54	(0.75–16.32)	0.11	31.52	(24.35–38.69)	0.47
Model 3				29.84	(18.51–41.16)	0.24	8.53	(0.70–16.37)	0.11	30.28	(22.94–37.63)	0.45
TAT												
Model 1	(Reference)			1.55	(0.10–3.01)	0.10	1.53	(0.52–2.54)	0.17	4.14	(3.21–5.07)	0.50
Model 2				1.86	(0.66–3.06)	0.12	1.02	(0.19–1.85)	0.11	3.56	(2.80–4.33)	0.43
Model 3				1.74	(0.55–2.92)	0.11	0.97	(0.15–1.79)	0.11	3.26	(2.49–4.03)	0.40
**Low hs-CRP group**												
PCF												
Model 1	(Reference)			12.94	(1.80–24.08)	0.10	15.48	(10.07–20.90)	0.26	31.79	(25.10–38.47)	0.42
Model 2				11.20	(0.75–21.65)	0.09	13.92	(8.80–19.04)	0.23	29.45	(23.17–35.72)	0.39
Model 3				10.21	(-0.36–20.78)	0.08	14.02	(8.87–19.18)	0.23	30.11	(23.76–36.47)	0.40
TAT												
Model 1	(Reference)			2.85	(1.47–4.22)	0.17	2.14	(1.47–2.81)	0.28	4.25	(3.43–5.08)	0.45
Model 2				2.72	(1.57–3.86)	0.17	1.69	(1.13–2.25)	0.22	3.80	(3.12–4.49)	0.40
Model 3				2.49	(1.34–3.64)	0.15	1.67	(1.11–2.23)	0.22	3.74	(3.05–4.43)	0.40

Model 1: Unadjusted.

Model 2: Adjusted for age and sex.

Model 3: Adjusted for age, sex, HTN, DM, Hyperlipidemia, smoking, alcohol drinking, and exercise.

Abbreviations as list in Table 1.

Table 3 presents the results of the ordered probit regression stratified by hs-CRP subgroup. Compared with the multiple linear regression, the ordered probit regression had similar results, except for the following differences: For the moderate/high hs-CRP subgroup, the correlation between NAFLD and TAT was greater than that between TAT and AO (standardized *β* = 3.62, NAFLD; standardized *β* = 2.99, AO); infra-additive effects were observed in both hs-CRP subgroups.

**Table 3:** Results of the ordered probit regression stratified by hs-CRP subgroup.

Variables	NAFLD (−) AO (−)	NAFLD (−) AO (+)			NAFLD (+) AO (−)			NAFLD (+) AO (+)		
		β	(95% CI)	Std. β	β	(95% CI)	Std. β	β	(95% CI)	Std. β
**Moderate/high hs-CRP group**										
PCF tertile										
Model 1	(Reference)	1.32	(0.86–1.78)	5.58	0.44	(0.14–0.75)	2.85	1.28	(0.99–1.57)	8.63
Model 2		1.45	(0.97–1.93)	5.91	0.41	(0.10–0.72)	2.59	1.28	(0.98–1.58)	8.45
Model 3		1.47	(0.99–1.96)	5.95	0.42	(0.11–0.73)	2.62	1.26	(0.96–1.57)	8.11
TAT tertile										
Model 1	(Reference)	0.46	(0.03–0.89)	2.08	5.87	(0.03–0.89)	4.00	1.15	(0.86–1.44)	7.76
Model 2		0.77	(0.28–1.25)	3.11	5.58	(0.28–1.25)	3.75	1.35	(1.03–1.67)	8.18
Model 3		0.75	(0.26–1.24)	2.99	5.60	(0.26–1.24)	3.61	1.29	(0.96–1.62)	7.60
**Low hs-CRP group**										
PCF tertile										
Model 1	(Reference)	0.70	(0.23–1.18)	2.88	0.72	(0.48–0.96)	5.87	1.35	(1.04–1.66)	8.58
Model 2		0.69	(0.20–1.18)	2.76	0.71	(0.46–0.95)	5.58	1.37	(1.05–1.68)	8.49
Model 3		0.68	(0.18–1.18)	2.65	0.71	(0.46–0.96)	5.60	1.41	(1.08–1.73)	8.55
TAT tertile										
Model 1	(Reference)	1.00	(0.52–1.49)	4.05	0.82	(0.57–1.06)	6.59	1.36	(1.05–1.67)	8.66
Model 2		1.38	(0.85–192)	5.04	0.90	(0.63–1.17)	6.59	1.62	(1.28–1.96)	9.35
Model 3		1.32	(0.78–1.87)	4.75	0.90	(0.63–0.17)	6.51	1.64	(1.29–1.98)	9.25

Model 1: Unadjusted.

Model 2: Adjusted for age and sex.

Model 3: Adjusted for age, sex, HTN, DM, Hyperlipidemia, smoking, alcohol drinking, and exercise.

Abbreviations as list in Table 1.

## Discussion

We showed a significant association between PCF/TAT and NAFLD/AO phenotypes in the retrospective cross-sectional study. We observed that the highest risk occurred in both abnormalities groups, and the second highest risk occurred in the AO-only group. Subjects with AO had a significantly increased risk of PCF or TAT compared to those with NAFLD. Notably, the magnitude of the associations between PCF/TAT and NAFLD/AO varied by the level of systemic inflammatory marker (hs-CRP level). Compared with low hs-CRP level, the magnitude of the associations were alleviated for NAFLD and PCF/TAT but enhanced for AO and PCF/TAT. Future studies are warranted to clarify the role of hs-CRP on the mechanism between PCF/TAT and NAFLD/AO. We suggested that people with AO and NAFLD must be more careful about changes in PCF and TAT. Also, PCF and TAT can be assessed by conveniently measuring waist circumference (AO). Regular measurement of WC could be a convenient way to monitor peri-cardiovascular fat (PCF and TAT), especially for PCF.

There were some inconsistent results between the multiple linear and ordered probit models. Among the NAFLD/AO phenotypes, the linear model compared the mean PCF and TAT, and the probit model compared the PCF and TAT tertiles. We think that the different statistical properties between the mean and the tertiles led to inconsistent results. Furthermore, we observed both supra-additive and infra-additive effects, but these non-additive effects might be statistical interactions. Future studies are warranted to examine the biological interactions between NAFLD and AO. MDCT, which can be used to measure EAT and TAT, provides reliable and robust modality for assessing the visceral abdominal fat, accurately and non-invasively^4,9,25^. Prior work suggests that EAT and TAT contribute to systemic inflammation and play an independent role in the pathogenesis of atherosclerosis and left ventricular (LV) structure or functional deterioration, especially strongly correlated with EAT, coronary calcium deposit, and LV remodeling^5,6,10,12,26–30^. In past decades, CAC assessment performed using MDCT has been well accepted clinically in preventive medicine for risk stratification in asymptomatic subjects^31,32^. The acquired MDCT data could also provide the opportunity to generate and calculate three-dimension (3D) volume-based regional-specific adipose tissue burden in the thorax. However, the cost of and radiation exposure required in MDCT are still worth noting. The safety, convenience, and predictive value of various anthropometric measurements for PCF and TAT require exploration.

Anthropometric measurements of WC are clinically convenient and can predict AO^8^. AO is strongly associated with hs-CRP, cardiovascular morbidity is associated with age^33,34^, and higher hs-CRP levels could predict NAFLD^29,30^. NAFLD is strongly linked to obesity, with a reported prevalence as high as 80% in obese patients^8,35^. AO has been consistently associated with NAFLD in 60–95% of cases, while AO assessed using the WC/hip ratio or WC is strongly related to the prevalence of NAFLD^36,37^. Research led by Dr Rosa Lombardi showed that 323 Italian patients with lean-NAFLD with increased levels of waist fat can in fact be at greater risk than obese patients with NAFLD^38^.

Previous studies have proven that the severity of NAFLD is strongly associated with the WC of participants, not only on the obesity of individuals^38,39^. In addition, Rosito et al. and Schlett et al. found that EAT and TAT were linearly related to WC^4,25^. Our study is consistent with these previous findings; after adjusting for known CVD risk factors such as age, chronic diseases, and lifestyle habits, patients with both NAFLD and AO had a synergistically increased risk of PCF and TAT. Moreover, after making the same adjustments, participants with AO only had a significantly higher risk of PCF and TAT than those with NAFLD only, possibly mediated by hs-CRP.

EAT which is a two-dimensional measure-plays a role in the early phases of asymptomatic atherosclerosis via several mechanisms, including inflammation, oxidative stress, and lipotoxicity^26,29,30^. Many studies have discussed the relationship between EAT and other body fat distribution and cardiovascular risk^27,28,32^. This paper provides prognostic information and provide a better prediction and stratification of CAD^40^. In a general community population (n = 2238), Meng et al. found that EAT is strongly associated with NAFLD after adjusting for other cardiovascular risk factors (OR [95% CI], 1.407 [1.117, 1.773])^24^. In fact, PCF and TAT are anatomically and biochemically distinguished from EAT^26–28^. Recently, 3D CT-measured EAT and TAT have been identified as novel risk markers for CVD^6,41^. To date, EAT plays a key role in CT-measured adipose tissue burden in CVD. This is only practical for ECG-gated cardiac CT. For large amounts of non-gated enhanced and non-enhanced chest CT studies in clinical practice, it is difficult to identify the thin pericardium without blurring artifacts due to heartbeats. Therefore, we used novel method to separate and quantify PCF and TAT and further reported a strong link between both PCF/TAT and NAFLD/ AO phenotypes after adjustment for all potential clinical confounders. AO had a stronger effect on visceral fat burden than NAFLD. We also observed that visceral fat was independently associated with hs-CRP after controlling for factors, which is crucial in recognizing again that MDCT-based visceral fat quantification may be predicted by AO/NAFLD component through mediation by systemic inflammation (hs-CRP)^21^.

In this study, participants with AO and without NAFLD were generally older and had higher blood pressure and fasting plasma glucose levels than subjects with NAFLD and without AO. Previous studies have closely associated AO with high blood pressure and insulin resistance, which are the factors that induce cardiovascular disease^42,43^.

There are several possible reasons for the weak association between TAT and inflammation markers in the AO/NAFLD phenotype. First, the pathogenesis of shifting from peripheral to central fat patterning may be induced according to the location and distribution of various adipose tissues; PCF and TAT are located in different heart areas. While PCF was observed combined with adipose tissue inside the pericardial sac surrounding the coronary arteries and surrounding pericardium. TAT is located at the area close to the thoracic aorta rather than coronary arteries^6^. Second, it has been reported that multiple markers of inflammation and oxidative stress correlate with PCF and TAT, such as interlukin-6 (IL-6) and CRP^29,30^. These mechanisms may help explain our finding regarding the differential influence and impact of hs-CRP on PCF compared to that of TAT.

There are some potential limitations to our study. First, our results might have varied according to the definition of WC (modified NCEP-ATP III for Taiwanese)^44^. Whether these results are applicable to ethnicities outside of Taiwanese and other East Asians is unknown. Second, a higher prevalence rate was found in Taiwan (11.5–52%)^18^. It was as high as 80% in participants who were overweight or obese^45^. In this study, the mean BMI was overweight, which resulted in higher prevalence of NAFLD (60.1%), this could have affected the relationship between NAFLD and PCF/TAT. Third, we also need to acknowledge the lack of assessment of proinflammatory cytokines, adipokines, or hormones (such as IL-6, or estrogen) in the current study. We were unable to further examine the role of proinflammatory cytokines in the mediating atherosclerotic pathophysiology underlying excessive visceral adiposity^46^. Fourth, determining the history of alcohol intake solely via a self-reported questionnaire might have introduced bias into the study. Fifth, abdominal ultrasound detects only moderate/severe hepatic steatosis. We use the double-blind progress of the operating physician to confirm the examination results, but in the future, we still need to use CT for liver fat confirmation. Finally, cross-sectional studies are unlikely to infer causality from the associations described.

In this health-screening population, PCF and TAT were significantly higher in participants with both NAFLD and AO abnormities, and this was higher in participants with AO-only than in those with NAFLD-only after adjusting for confounding factors. Higher hs-CRP levels were found to have a stronger association with PCF in the AO abnormity group. Limited information is available on the exact roles of NAFLD and AO as risk factors for PCF and TAT in previous studies. With the data from our study, we hope to determine the underlying pathogenesis related to the association of NAFLD and AO with TAT and PCF so that we can reduce the WC and occurrent of NAFLD and decrease in inflammation markers (such as hs-CRP) by establishing a practical lifestyle and appropriate drug treatment, consequently preventing and reducing the occurrence and risk of PCF and TAT, ultimately decreasing the prevalence of CAD.

## Methods

### Ethics

The study protocol was evaluated and approved by the Human Research Ethics Committee of Mackay Memorial Hospital (project research number 18MMHIS137, October 15, 2018) and was in accordance with the Helsinki Declaration of 1975. Because of the retrospective study design, the need for informed consent was waived by the Institutional Review Board of the Human Research Ethics Committee of Mackay Memorial Hospital. All study participants were de-identified during the data analysis.

### Study design and study population

**Figure 1:**
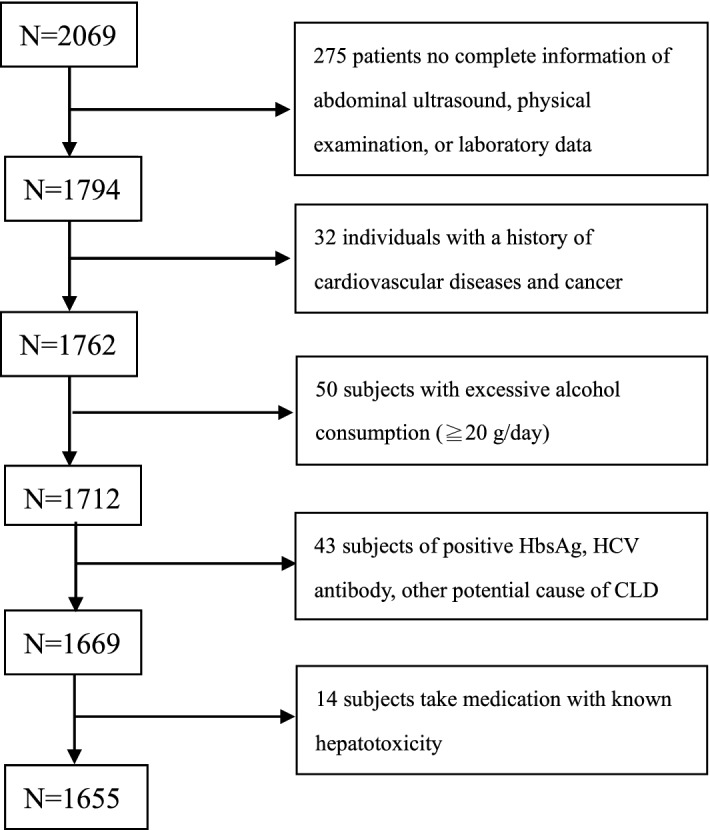
Flow chart of enrolled participants meet the requirements.

This cross-sectional study retrospectively enrolled 2,069 adults who participated in a health-screening program at a tertiary medical center in Taipei, Taiwan, between 2005 and 2009. MDCT was performed to assess the PCF and TAT of all participants. Participants were excluded if they had any of the following issues: (1) incomplete information on abdominal ultrasound, waist circumference, or missing information about metabolic components; (2) self-reported history of heart attack or coronary artery disease including acute myocardial infarction, angina, or congestive heart disease; (3) excessive alcohol consumption (≥ 20 g/day); (4) presence of serological evidence of viral hepatitis or other chronic liver disease; and (5) current use of known hepatoxic medicine (e.g., statins or fibrates, estrogen, tamoxifen, diltiazem, and valproate) during the previous year (Fig. 1). A total of 1655 patients (age: 49.4 ± 9.8 years; 29.7% female) were included in the analysis. A detailed physical examination and thorough review of the baseline characteristics and medical history were performed using structured questionnaires. A history of hypertension (HTN) was defined as systolic blood pressure ≥ 140 mmHg, diastolic blood pressure ≥ 90 mmHg, or a previous diagnosis of HTN with current medication. A history of diabetes mellitus (DM) was defined as a fasting glucose level ≥ 126 mg/dL or the current use of any diabetic medication for treating previously diagnosed DM. A history of hyperlipidemia was defined as a fasting total cholesterol level ≥ 200 mg/dL, triglyceride level ≥ 150 mg/dL, or the current use of any lower lipid medication for treating previously diagnosed hyperlipidemia.

### Baseline anthropometric measurements

Baseline characteristics and anthropometric measurements, including age, body height, body weight, body mass index (BMI), and WC were recorded. WC was measured at the midpoint between the lowest rib and upper point of the iliac crest and at the end of normal expiration. Standardized sphygmomanometer cuff-defined resting blood pressure values were measured while resting. Anthropometric measurements were recorded by trained nurses who were blinded to the patients’ information in a laboratory center.

### Laboratory data acquisition and analysis

A Hitachi 7170 Automatic Analyzer (Hitachi Corp., Hitachinaka Ibaraki, Japan) was used to measure the levels of fasting glucose (hexokinase method), total cholesterol (TC), lipid profiles (including low-density and high-density lipoprotein cholesterol [LDLC and HDL-C, respectively)]; homogenous enzymatic colorimetric assay, triglyceride (TG), aspartate aminotransferase (AST), alanine aminotransferase (ALT), hepatitis B surface antigen, and hepatitis C virus antibody. Hs-CRP levels were determined using a highly sensitive latex particle-enhanced immunoassay (Elecsys 2010; Roche, Mannheim, Germany)^6^. Venous blood samples were taken from all subjects before 10 AM after a 12-h overnight fast. All laboratory measurements were performed using standard laboratory methods.

### Diagnosis of NAFLD and AO

Abdominal ultrasonography was performed by two experienced gastroenterologists who were blinded to the laboratory and clinical details of the subjects at the time of the procedure. If different results were found, a third doctor was asked to perform the test while blinded to test results and patient information. Abdominal ultrasonography (Acuson, Sequoia 512, Siemens, Mountain View, CA, USA) was used to diagnose fatty liver. According to the Asia-Pacific Working Party on NAFLD and Chinese Association for the Study of Liver Disease (CASLD), diagnosis of fatty liver disease was based on the presence of at least two of the following three abnormal findings: (1) diffusely increased liver near-field ultrasound echo (“bright liver”); (2) liver echo greater than kidney echo, and (3) vascular blurring and gradual attenuation of far-field ultrasound echo^47,48^.

Abdominal obesity (AO) was defined as waist circumference ≥ 90 cm for men and 80 cm for women according to the classification of the National Cholesterol Education Panel of the National Treatment Program for Adults III (NCEP-ATP III) was used with specific cutoff points for the Taiwanese population for AO^44^.

### Measurements of PCF and TAT

**Figure 2:**
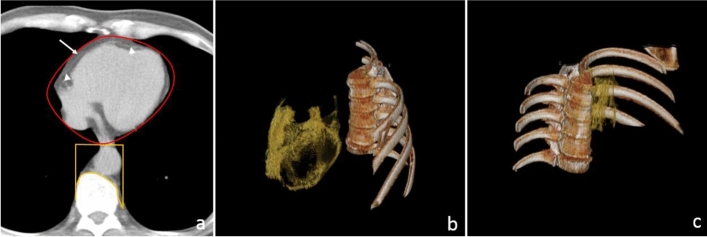
Multidetector computed tomography (MDCT) demonstrated visceral adiposity measures including pericardial and thoracic peri-aortic fat tissue. The pericardial fat was defined as the volume-based burden of total adipose tissue located within the pericardial sac and adipose tissue outside the pericardium, but in the paracardial region of the anterior mediastinum and anterior to the esophagus and thoracic descending aorta was defined as the fat between the heart and the pericardium (arrowhead) plus paracardial adipose tissue (arrow) which was close and outside pericardium (Region of interest (ROI) with red border) as well as the thoracic peri-aortic fat was defined as the fat surrounding the thoracic aorta (ROI with yellow border), as shown in axial view (**a**). 3D reconstruction of pericardial adipose tissue (**b**) and thoracic peri-aortic adipose tissue (**b**). *Orange color regions indicate visceral fat tissue.

The visceral adipose tissues of PCF and TAT were quantified using MDCT with a dedicated workstation (Aquarius 3D Workstation). A semi-automatic segmentation technique was developed for quantifying fat volume. We manually traced the region of interest and defined the fat tissue as pixels within a window of − 195 to − 45 HU and a window center of − 120 HU. PCF was defined as the volume-based burden of total adipose tissue located within the pericardial sac and adipose tissue outside the pericardium, but in the paracardial region of the anterior mediastinum and anterior to the esophagus and thoracic descending aorta (Fig. 2a, b). TAT tissue was defined as the total adipose tissue volume surrounding the thoracic aorta (as periaortic fat), which extends 67.5 mm from the level of bifurcation of the pulmonary arteries (Fig. 2a, c) with cranial-caudal coverage of the thoracic aorta^6^.

### Statistical analysis

We divided NAFLD/AO phenotypes into four groups: (1) NAFLD (−) and AO (−) (2) NAFLD (−) and AO (+) (3) NAFLD (+) and AO (−) (4) NAFLD (+) and AO (+), according to the presence/absence of NAFLD diagnosed via ultrasonography and the presence/absence of AO based on WC in an apparently healthy Taiwanese population. The hs-CRP level was dichotomized into a moderate/high risk group (≥ 0.1 mg/dL) and low risk group (< 0.1 mg/dL). Besides using continuous PCF and TAT values, we also created PCF tertile (< 59.54 ml, 59.54–83.43 ml, and ≥ 83.43 ml) and the TAT tertile (< 4.65 ml, 4.65–7.73 ml, and ≥ 7.73 ml) for analysis. The baseline clinical, demographic, anthropometric, and laboratory data are presented as the mean ± SD for continuous variables and n (%) for categorical variables. One-way analysis of variance (one-way ANOVA) was used to compare the mean PCF or mean TAT between the four phenotypes. The chi-squared test or Fisher's exact test were used to examine the association between the PCFor TAT tertile and NAFLD/AO phenotype. For multiple comparisons, we conducted Bonferroni correction to handle the inflation of type I error.

We performed a subgroup analysis based on hs-CRP levels and conducted multivariate analysis for each subgroup. Multiple linear regression and ordered probit models were used for multivariate analysis. We selected variables of clinical importance and statistical significance in the regression models, including age, hypertension, diabetes, hyperlipidemia, cigarette smoking, alcohol consumption, and exercise. All analyses were conducted using SPSS (IBM Corp., Armonk, NY, USA) for Windows. A two-sided *p* value < 0.05 was considered statistically significant.

### Institutional review board statement

The study protocol was evaluated and approved by the Human Research Ethics Committee of MacKay Memorial Hospital (project research number 18MMHIS137, 15 Oct 2018).

### Informed consent statement

Informed consent was waived from our institutional board review due to retrospective study design. All study participants were de-identified during data analysis.

## Supplementary Information

Supplementary Figure 1.

## Data Availability

No additional data are available.
