# Supplementary material for: Pericardial fat, thoracic peri-aortic adipose tissue, and systemic inflammatory marker in nonalcoholic fatty liver and abdominal obesity phenotype
Source: Sci Rep. 2022 Feb 4;12:1958. doi: 10.1038/s41598-022-06030-z (PMC8816900; doi:10.1038/s41598-022-06030-z)
Supplement: Supplementary file 1 — Supplementary Figure 1. [file 41598_2022_6030_MOESM1_ESM.pdf]

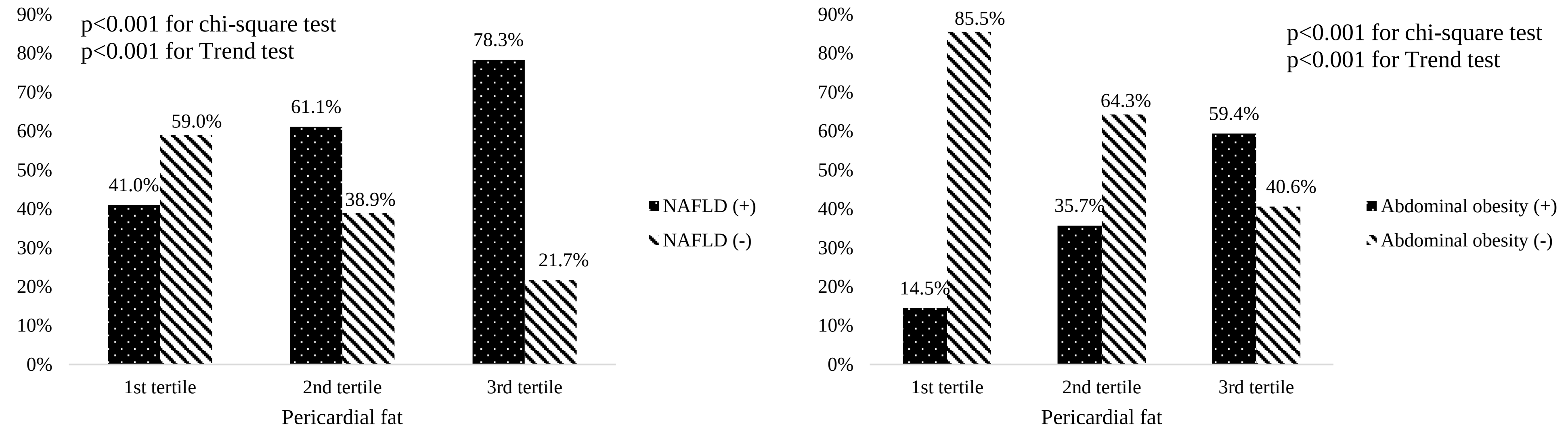

a. Pericardial fat

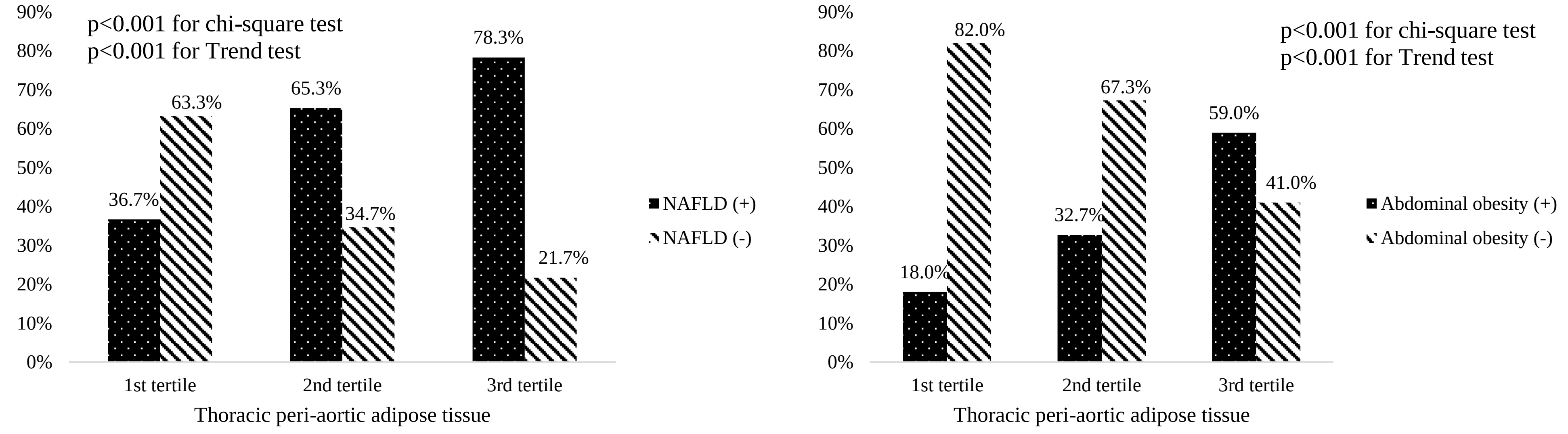

b. Thoracic peri-aortic adipose tissue

Supplement figure 1: Prevalence of NAFLD and AO according to the visceral fat burden groups a: PCA; b: TAT.
